# A comprehensive analysis of Fanconi anemia genes in Chinese patients with high-risk hereditary breast cancer

**DOI:** 10.1007/s00432-023-05236-6

**Published:** 2023-08-11

**Authors:** Qiao-Yan Zhu, Pu-Chun Li, Yi-Fan Zhu, Jia-Ni Pan, Rong Wang, Xiao-Lin Li, Wei-Wu Ye, Xiao-Wen Ding, Xiao-Jia Wang, Wen-Ming Cao

**Affiliations:** 1 Department of Breast Medical Oncology, Zhejiang Cancer Hospital, Hangzhou, 310022 People’s Republic of China; 2 The Second Clinical Medical College of Zhejiang, Chinese Medical University, Hangzhou, 310053 People’s Republic of China; 3 Wenzhou Medical University, Wenzhou, 325035 China; 4 Department of Tumor Surgery, Zhejiang Cancer Hospital, Hangzhou, 310022 People’s Republic of China

**Keywords:** Fanconi anemia genes, Breast cancer, Mutation, Susceptibility, Prognosis

## Abstract

**Background:**

Four Fanconi anemia (FA) genes (*BRCA1*, *BRCA2, PALB2* and *RAD51C*) are defined as breast cancer (BC) susceptibility genes. Other FA genes have been inconsistently associated with BC. Thus, the role of other FA genes in BC should be explored in specific populations.

**Methods:**

Mutations in 16 FA genes were screened with a 98-gene panel sequencing assay in a cohort of 1481 Chinese patients with high-risk hereditary BC. The association between mutations and clinicopathological characteristics as well as prognosis was analyzed. The risk of BC in carriers of FA gene mutations was assessed in the Genome Aggregation Database and the Westlake Biobank for Chinese cohort.

**Results:**

A total of 2.57% (38/1481) BC patients were identified who had 12 other FA gene germline mutations. Among them, the most frequently mutated gene was *FANCA* (8/1481, 0.54%). These 38 patients carried 35 distinct pathogenic/likely pathogenic variants, of which 21 were novel. We found one rare *FANCB* deleterious variant (c.1327-3dupT) in our cohort. There was a statistically significant difference in lymph node status between FA gene mutation carriers and non-carriers (p = 0.041). We observed a trend that mutation carriers had larger tumor sizes, lower estrogen receptor (ER) and progesterone receptor (PR) positivity rates, and lower 3.5-year invasive disease-free survival (iDFS) and distant recurrence-free survival (DRFS) rates than non-carriers (tumor size > 2 cm: 51.43% vs. 45.63%; ER positivity rates: 51.43% vs. 60.81%; PR positivity rates: 48.57% vs. 55.16%; 3.5-year iDFS rates: 58.8% vs. 66.7%; 3.5-year DRFS rates: 58.8% vs. 68.8%). The frequency of the mutations in *FANCD2*, *FANCM* and *BRIP1* trended to be higher among BC cases than that in controls (*p* = 0.055, 0.08 and 0.08, respectively).

**Conclusion:**

This study comprehensively estimated the prevalence, clinicopathological characteristics, prognosis and risk of BC associated with deleterious variants in FA genes in Chinese high-risk hereditary BC patients. It enriches our understanding of the role of FA genes with BC.

## Introduction

Breast cancer (BC) is the most common malignancy that affects women worldwide. BC is highly associated with genetic factors (Castéra et al. 2014; Sung et al. 2021; Tung et al. 2016). Germline variants in 13 susceptibility genes have been shown to be related to the tumorigenesis and risk of BC. These genes are *BRCA1*, *BRCA2*, *TP53*, *ATM*, *RAD51C*, *RAD51D*, *PALB2*, *CHEK2*, *NF1*, *BARD1*, *PTEN*, *STK11* and *CDH1* (Dorling et al. 2021; Easton et al. 2015). Among them, *BRCA1* and *BRCA2* were the first genes reported to be associated with an increased risk of breast and ovarian cancer (Szabo et al. 1995; Wooster et al. 1994). A prospective cohort study showed a cumulative BC risk of 72% for *BRCA1* carriers and 69% for *BRCA2* carriers by age 80 (Kuchenbaecker et al. 2017). In a meta-analysis, the estimated relative risk of a *PALB2* mutation in BC was approximately 5.3 (Easton et al. 2015). The estimated relative risk of *RAD51C* in BC was 1.99, with an estimated cumulative risk of 21% for the occurrence of BC up to 80 years old (Yang et al. 2020). *FANCS*/*BRCA1*, *FANCD1*/*BRCA2*, *FANCN*/*PALB2* and *FANCO*/*RAD51C* are Fanconi anemia (FA) genes (Fang et al. 2020). Other than these, 18 genes have been described as FA genes: *FANCA*, *FANCB*, *FANCC*, *FANCD2*, *FANCE*, *FANCF*, *FANCG*/*XRCC9*, *FANCI*, *FANCJ*/*BRIP1*, *FANCL*/*PHF9*, *FANCM*, *FANCP*/*SLX4*, *FANCQ*/*ERCC4*, *FANCR*/*RAD51*, *FANCT*/*UBE2T*, *FANCU*/*XRCC2*, *FANCV*/*REV7* (Bluteau et al. 2016) and *FANCW*/*RFWD3* (Knies et al. 2017; Nalepa et al. 2018). The genetic susceptibility of 17 of these genes other than *FANCW* to BC has been much studied, but the results are conflicting and await further exploration (Gianni et al. 2022).

FA is an uncommon genetic disorder characterized by progressive aplastic anemia, congenital malformations and tumor susceptibility (Mamrak et al. 2017). FA gene products are involved in the FA-BRCA pathway, coordinating nucleolytic incision, translesion DNA synthesis and homologous recombination (HR), and they play a key role in DNA damage, particularly in DNA interstrand cross-link (ICL) repair (Kim et al. 2012; Zhang et al. 2014). In addition, FA proteins protect genomic stability by regulating the cell cycle checkpoint and replication fork remodeling (Badra Fajardo et al. 2022). FA pathway-deficient tumor cells are more sensitive to the DNA ICL inducer cisplatin after inhibition of the FA pathway (Jacquemont et al. 2012). Tumors with germline mutations in FA genes encoding HR proteins are sensitive to DNA damaging agents including cisplatin and Poly (ADP-ribose) polymerase (PARP) inhibitors due to accumulated DNA lesions (Cong et al. 2021; Ray Chaudhuri et al. 2016; Simoneau et al. 2021). This suggests that disease-causing pathogenic germline variants in FA genes may be important therapeutic targets that can get benefit from targeted alternative DNA repair pathways.

The association between FA genes (except *BRCA1, BRCA2*, *PALB2* and *RAD51C*) that have not been confirmed as BC susceptibility genes has been less studied in the Chinese population. Additionally, whether pathogenic variants in FA genes have prognostic impact on clinical outcomes in patients with BC is unknown. To explore the role of FA genes in BC, we studied the mutation profile of FA genes in 1481 patients with high-risk hereditary BC and investigated whether the presence of FA gene mutations affected the clinicopathological characteristics and outcomes of BC patients. We also explored the risk of BC by comparing the FA variants identified in our cohort with non-cancer patients in the Genome Aggregation Database (GnomAD) East Asian cohort and the Westlake Biobank for Chinese (WBBC) cohort.

## Materials and methods

### Patients

We conducted a prospective cohort study including 1481 cases with hereditary high-risk BC who underwent genetic counseling/testing at the Zhejiang Cancer Hospital from February 2008 to April 2022 to explore the role of FA genes in BC. Patients were enrolled based on the National Comprehensive Cancer Network guidelines for genetic/familial high-risk assessment on breast, ovarian and pancreatic cancer (Daly et al. 2021). High-risk hereditary BC patients fulfilled at least one of the following criteria: (1) diagnosed with BC at age ≤ 40 years; (2) diagnosed with triple-negative breast cancer (TNBC) at ≤ 50 years; (3) diagnosed with bilateral or ipsilateral multi-focal BC; (4) male BC; (5) having a minimum of one first- or second-degree relative who had BC, ovarian cancer, pancreatic cancer or distant metastatic prostate cancer; individuals with ovarian cancer and/or pancreatic cancer. Clinical information including clinicopathological data, outcome variables and familial history of cancer was collected from medical records and/or by telephone follow-up. The study was approved by the Research and Ethics Committee of Zhejiang Cancer Hospital. Written informed consent was obtained from all subjects.

### FA gene variants

DNA samples were isolated from peripheral blood samples of BC patients with the QIAamp DNA Blood Mini kit (Qiagen). A panel (Yang et al. 2023) covering whole exons of 98 genes was used to identify variants in FA genes. Details of the DNA sequencing and bioinformatic analysis have been published previously (Zhu et al. 2022). Briefly, all samples were diluted and pooled in a HiSeq X-Ten (Illumina) for multiplexed sequencing.

The variants were interpreted and filtered according to the American College of Medical Genetics and Genomics Standards and Guidelines for the Interpretation of Sequence Variants. The evidence was based on databases and predictive software such as ClinVar (https://www.ncbi.nlm.nih.gov/) and the Human Gene Mutation Database (http://www.hgmd.org/). Only variants classified as pathogenic or likely pathogenic were included.

### GnomAD and WBBC analysis

The GnomAD East Asian, non-cancer subpopulation (v.2.1.1, http://www.gnomad-sg.org/) and the WBBC cohort (GRCh37, https://wbbc.westlake.edu.cn/index.html) were used as control populations. Variants predicted to be loss-of-function in FA genes were exported to test the associations between FA genes and BC risk.

### Statistical analysis

Variables included age at diagnosis, personal and family history of BC, personal and family history of ovarian cancer, tumor size, lymph node status, pathological type, nuclear grade (I, II and III), vascular invasion, estrogen receptor (ER) and progesterone receptor (PR) status, HER2 receptor status, age of menarche and menopause and BMI. Continuous variables were analyzed with a *t* test. Comparison of categorical variables was conducted using the Chi-square test or Fisher’s exact test.

Follow-up started at the time blood was drawn. The latest date of follow-up was when patients visited the physician or received telephone call from us at the last time. Distant recurrence-free survival (DRFS) was defined as the time from the date of surgery to distant recurrence or death from any cause. Invasive disease-free survival (iDFS) was measured from the date of surgery to the date of first occurrence of ipsilateral invasive breast tumor recurrence, local/regional invasive BC recurrence, distant recurrence, death attributable to any cause, contralateral invasive BC or a second primary non-breast invasive cancer. The definition of loss to follow-up was event-free patients with a follow-up period of more than 5 years who were out of touch for over 1.5 years or event-free patients with follow-up period of within 5 years who were out of touch for more than 1 year. A total of 21% of patients were lost to follow-up until April 2022.The Kaplan–Meier method was used to assess DRFS and iDFS. Associations between FA genes and BC risk were estimated by logistic regression.

Statistical significance was defined as a two-tailed *p* value < 0.05. All analyses were performed using SPSS Statistics 25.0 software (IBM, Armonk, NY).

## Results

### Prevalence of FA gene germline mutations

Genetic testing was performed in 1481 patients with high-risk hereditary BC. A total of 313 patients were identified as carrying at least one of 13 BC susceptibility gene mutations, and 38 patients carried 35 distinct pathogenic/likely pathogenic variants in 12 FA genes (*BRCA1*/*2*, *PALB2* and *RAD51C* were not included). Mutations in *FANCF*, *FANCR*/*RAD51*, *FANCT*/*UBE2T* and *FANCU*/*XRCC2* were not found in this cohort. Among the 38 FA gene mutation carriers, the most frequently mutated gene was *FANCA* (8/1481, 0.54%), and other mutations found were in *FANCD2* (6/1481, 0.41%), *FANCM* (5/1481, 0.34%), *BRIP1* (5/1481, 0.34%), *FANCC* (4/1481, 0.27%), *FANCI* (2/1481, 0.14%), *FANCL* (2/1481, 0.14%), *FANCP*/*SLX4* (2/1481, 0.14%), *FANCQ*/*ERCC4* (1/1481, 0.07%), *FANCE* (1/1481, 0.07%), *FANCG* (1/1481, 0.07%) and *FANCB* (1/1481, 0.07%). Seven *FANCA* deleterious variants were detected in eight patients, including 2 (2/8, 25%) frameshift, 2 (2/8, 25%) stop-gain, 2 (2/8, 25%) splicing and 2 (2/8, 25%) missense variants. Four (4/8, 50%) novel *FANCA* variants were identified: c.3393dupT (p.Ala1132Cysfs*83, *n* = 1), c.1715 + 1G > C (*n* = 1), c.3342dupT (p.Glu1115Ter, *n* = 1) and c.1287delT (p.Ala430ArgfsTer96, *n* = 1). Furthermore, 17 novel variants in other FA genes was identified: *BRIP1* c.3182_3189delACACATCG (p.Asn1061Ilefs*17, *n* = 1), *BRIP1* c.3223delT (p.Ser1075Hisfs*3, *n* = 1), *FANCB* c.1327-3dupT (*n* = 1), *FANCC* c.844-1G > A (*n* = 1), *FANCC* c.887_890dupAGAT (p.Met297Ilefs*78, *n* = 1), *FANCD2* c.1991_1992insA (p.Phe664Leufs*12, *n* = 1), *FANCD2* c.1656 + 2 T > A (*n* = 1), *FANCD2* c.783 + 1G > A (*n* = 1), *FANCD2* c.2155G > T (p.Glu719Ter, *n* = 1), *FANCI* c.1954_1955dupTC (p.Thr653Ter, *n* = 1), *FANCI* c.2889 + 1G > C (*n* = 1), *FANCL* c.857 T > G (p.Leu286Ter, *n* = 1), *FANCL* c.555 + 1G > T (*n* = 1), *FANCM* c.4515 + 1G > C (*n* = 2), *FANCM* c.170_189delTGCTTGTCGCGGCGTACGAG (p.Leu57Cysfs*2, *n* = 1), *FANCP*/*SLX4* c.817C > T (p.Gln273Ter, *n* = 1) and *FANCP*/*SLX4* c.4481delG (p.Gly1494Alafs*13, *n* = 1). Notably, two patients carried two distinct variants: *FANCA* with *BRCA1* and *FANCD2* with *BRCA2*. One patient with three distinct mutations in *ATM*, *BLM* and *FANCA* was observed (see Table 1).

**Table 1:** The 35 Fanconi anemia (FA) gene mutations were identified in a cohort of 1481 patients with high-risk hereditary breast cancer

Gene	cDNA change	Exon	No. case	Amino acid change	Type of mutation	References
*BRIP1*	c.1776G > A	exon12	1	p.Trp592Ter	stop-gain	Reported on ClinVar
*BRIP1*	c.3182_3189delACACATCG	exon20	1	p.Asn1061Ilefs*17	frameshift	Novel
*BRIP1*	c.3223delT	exon20	1	p.Ser1075Hisfs*3	frameshift	Novel
*BRIP1*	c.409_410delAA	exon5	1	p.Lys137Valfs*4	frameshift	Reported on ClinVar
*BRIP1*	c.1A > G	exon2	1	p.Met1	start-loss	Reported on ClinVar
*FANCA*	c.1303C > T	exon14	2	p.Arg435Cys	missense	Reported on ClinVar
*FANCA*	c.3393dupT	exon34	1	p.Ala1132Cysfs*83	frameshift	Novel
*FANCA*	c.1715 + 1G > C	intron18	1		splicing	Novel
*FANCA*	c.4010 + 2 T > C	intron40	1		splicing	Reported on ClinVar
*FANCA*	c.3342dupT	exon33	1	p.Glu1115Ter	stop-gain	Novel
*FANCA*	c.3931_3932delAG	exon39	1	p.Ser1311Ter	stop-gain	Reported on ClinVar
*FANCA*	c.1287delT	exon14	1	p.Ala430ArgfsTer96	frameshift	Novel
*FANCB*	c.1327-3dupT	intron6	1		frameshift	Novel
*FANCC*	c.339G > A	exon4	2	p.Trp113Ter	stop-gain	Reported on ClinVar
*FANCC*	c.844-1G > A	intron8	1		splicing	Novel
*FANCC*	c.887_890dupAGAT	exon9	1	p.Met297Ilefs*78	frameshift	Novel
*FANCD2*	c.990-1G > A	intron12	1		splicing	(Kalb et al. 2007)
*FANCD2*	c.1991_1992insA	exon22	1	p.Phe664Leufs*12	frameshift	Novel
*FANCD2*	c.1656 + 2 T > A	intron18	1		splicing	Novel
*FANCD2*	c.1222C > T	exon15	1	p.Arg408Ter	stop-gain	Reported on ClinVar
*FANCD2*	c.783 + 1G > A	intron10	1		splicing	Novel
*FANCD2*	c.2155G > T	exon23	1	p.Glu719Ter	stop-gain	Novel
*FANCE*	c.598C > T	exon2	1	p.Arg200Cys	missense	Reported on ClinVar
*FANCG*	c.1066C > T	exon8	1	p.Gln356Ter	stop-gain	Reported on ClinVar
*FANCI*	c.1954_1955dupTC	exon20	1	p.Thr653Ter	stop-gain	Novel
*FANCI*	c.2889 + 1G > C	intron27	1		splicing	Novel
*FANCL*	c.857 T > G	exon11	1	p.Leu286Ter	stop-gain	Novel
*FANCL*	c.555 + 1G > T	intron7	1		splicing	Novel
*FANCM*	c.4515 + 1G > C	intron17	2		splicing	Novel
*FANCM*	c.1236_1237delCT	exon7	1	p.Tyr413Ter	frameshift	Reported on ClinVar
*FANCM*	c.81delC	exon1	1	p.Gly28Glufs*43	frameshift	Reported on ClinVar
*FANCM*	c.170_189delTGCTTGTCGCGGCGTACGAG	exon1	1	p.Leu57Cysfs*2	frameshift	Novel
*ERCC4*	c.2169C > A	exon11	1	p.Cys723Ter	stop-gain	Reported on ClinVar
*SLX4*	c.817C > T	exon4	1	p.Gln273Ter	stop-gain	Novel
*SLX4*	c.4481delG	exon12	1	p.Gly1494Alafs*13	frameshift	Novel

### Association between FA gene germline mutations and clinicopathological characteristics

According to the results of genetic testing, 35 patients (excluding the three patients carrying multiple different gene mutations) carrying one germline mutation in an FA gene (except *BRCA1/2*, *PALB2* and *RAD51C*) were included in the mutation group, and 1,133 patients who did not carry any mutations in BC susceptibility genes were the control group. The differences in clinicopathological characteristics between FA mutation carriers and non-carriers were compared (Table 2). There was a statistically significant difference in lymph node status in FA gene mutations carriers when compared to the control group (*p* = 0.041). Mutation carriers had a trend toward larger tumor sizes and lower ER/PR positivity rates than non-carriers (tumor sizes > 2 cm: 51.43% vs. 45.63%; ER positivity rates: 51.43% vs. 60.81%; PR positivity rates: 48.57% vs. 55.16%). However, a significant statistical difference in tumor size and ER/PR status and other was not observed between the FA gene mutation carriers and non-carriers.

**Table 2:** Comparison of clinicopathological characteristics between 35 patients carrying one Fanconi anemia (FA) gene (except *BRCA1*/*2*, *PALB2* and *RAD51C*) pathogenic/likely pathogenic variant and 1133 patients who did not carry a breast cancer susceptibility gene mutation

	Carriers	Non-carriers	*P* value
	*n* = 35 (%)	*n* = 1133 (%)	
Age at diagnosis (years)			
Mean (SD)	42.6(8.311)	42.10(10.214)	0.776
Median (p25, p75)	41.00 (35, 50)	40.00(35, 48)	
< = 40	17 (48.57)	624 (55.08)	0.446
> 40	18 (51.43)	509 (44.92)	
Personal history of breast cancer			
Yes	7 (20.00)	206 (18.18)	0.784
No	28 (80.00)	927 (81.82)	
Personal history of ovary cancer			
Yes	0 (0.00)	10 (0.88)	> 0.999
No	34 (100.00)	1109 (97.88)	
Family history of breast cancer			
Yes	11 (31.43)	387 (34.16)	0.737
No	24 (68.57)	746 (65.84)	
Family history of ovary cancer			
Yes	0 (0.00)	30 (2.65)	> 0.999
No	35 (100.00)	1103(97.35)	
Tumor size			
≤ 2 cm	14 (40.00)	508 (44.84)	0.795
> 2 cm	18 (51.43)	517 (45.63)	
Unknown	3 (8.57)	108 (9.53)	
Lymph nodes status			
N0	11 (31.43)	497 (43.87)	**0.041**
N1	11 (31.43)	328 (28.95)	
N2	10 (28.57)	128 (11.30)	
N3	3 (8.57)	125 (11.03)	
Unknown	0 (0.00)	55 (4.85)	
Pathological type			
Non-invasive carcinoma with good prognosis	1 (2.86)	40 (3.53)	0.517
Invasive ductal carcinoma	32 (91.43)	927 (81.82)	
Invasive lobular carcinoma	1 (2.86)	30 (2.65)	
Invasive special carcinoma with good prognosis	0 (0.00)	41 (3.62)	
Other types with poor prognosis	0 (0.00)	71 (6.27)	
Unknown and other	1 (2.86)	24 (2.12)	
Grade			
I	0 (0.00)	28 (2.47)	0.573
II	11 (31.43)	391 (34.51)	
III	15 (42.86)	358 (31.60)	
Unknown	9 (25.71)	356 (31.42)	
Vascular invasion			
Yes	6 (17.14)	237 (20.92)	0.845
No	29 (82.86)	872 (76.96)	
Unknown	0 (0.00)	24 (2.12)	
Estrogen receptor			
Positive	18 (51.43)	689 (60.81)	0.203
Negative	17 (48.57)	406 (35.83)	
Unknown	0 (0.00)	38 (3.35)	
Progesterone receptor			
Positive	17 (48.57)	625 (55.16)	0.31
Negative	18 (51.43)	468 (41.31)	
Unknown	0 (0.00)	40 (3.53)	
HER2/neu receptor			
Positive	10 (28.57)	296 (26.13)	0.704
Negative	21 (60.00)	745 (65.75)	
Unknown	4 (11.43)	92 (8.12)	
Menopause at onset			
Yes	7 (20.00)	205 (18.09)	0.938
No	24 (68.57)	819 (72.29)	
Unknown	3 (8.57)	95 (8.38)	
BMI			
Mean (SD)	22.68 (3.407)	22.65(3.044)	0.954
Age at menarche			
Mean (SD)	14.94(1.458)	14.50(1.618)	0.128
Age at menopause			
Mean (SD)	49.22(2.863)	49.19(4.936)	0.985

Bold value indicate *p*-value less than 0.05 is statistically significant

### Comparison of survival among FA gene variant carriers and non-carriers

**Fig. 1:**
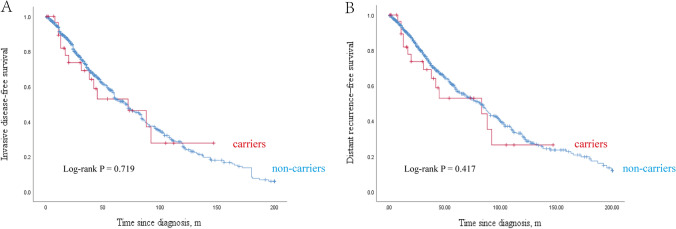
Kaplan–Meier invasive disease-free survival and distant recurrence-free survival among Fanconi anemia (FA) gene mutations carriers and non-carriers. Invasive disease-free survival (**A**) and distant recurrence-free survival (**B**)

After a median follow-up of 38 months (range 1–200 months), we compared the survival rate between FA gene mutation carriers and non-carriers (Fig. 1). There was a trend toward a difference in the 3-year iDFS and DRFS rates between carriers and non-carriers (3.5-year iDFS rates: 58.8% vs. 66.7%; 3.5-year DRFS rates: 58.8% vs. 68.8%), but there was no statistically significant difference between the two groups (*p* = 0.719 and 0.417 for iDFS and DRFS, respectively).

### FA gene mutations and BC risk

When comparing the mutation frequencies in FA genes in our cohort with those from the East Asian (non-cancer) GnomAD v.2.1 population, there was no association between the 11 FA genes identified in our cohort and BC risk (Table 3). The East Asian population in the GnomAD database contains Japanese, Korean and other ethnic groups. Therefore, we compared variant frequencies in the WBBC database (Table 3). Mutations in *FANCD2*, *FANCM* and *BRIP1* were more common in our cohort when compared to controls (without achieving statistical significance; *p* = 0.055, 0.08 and 0.08, respectively). There were no deleterious variants in *FANCB* in the East Asian population in GnomAD or in the WBBC. Thus, we could not estimate the difference in *FANCB* by logistic regression.

**Table 3:** Associations between pathogenic/likely pathogenic variants in Fanconi anemia (FA) genes and breast cancer risk in different clinical groups

Genes	Our cohort		GnomAD^a^		WBBC					
	Carriers	AF	Carriers	AF	Carriers	AF	OR (95% CI)^*^	P^*^	OR (95% CI)^**^	P^**^
*FANCA*	8	0.002700878	42	0.002883905	17	0.001897321	0.936 (0.439–1.998)	0.865	1.426 (0.614–3.311)	0.409
*FANCB*	1	0.00033761	0	0	0	0	–	–		–
*FANCC*	4	0.001350439	6	0.000910469	8	0.000892857	1.485 (0.418–5.268)	0.541	1.514 (0.455–5.035)	0.499
*FANCD2*	6	0.002025658	13	0.00128923	6	0.000669643	1.573 (0.597–4.146)	0.359	3.033 (0.977–9.419)	0.055
*FANCE*	1	0.00033761	3	0.000747164	5	0.000558036	0.452 (0.047–4.346)	0.491	0.605 (0.071–5.180)	0.646
*FANCG*	1	0.00033761	15	0.000815549	4	0.000446429	0.414 (0.055–3.133)	0.393	0.756 (0.084–6.770)	0.803
*FANCI*	2	0.000675219	17	0.00095812	6	0.000669643	0.704 (0.163–3.052)	0.639	1.008 (0.203–5.001)	0.992
*FANCL*	2	0.000675219	4	0.000804545	13	0.001450893	0.839 (0.154–4.587)	0.84	0.465 (0.105–2.061)	0.313
*FANCM*	5	0.001688049	7	0.001556431	5	0.000558036	1.085 (0.344–3.425)	0.889	3.032 (0.877–10.487)	0.08
*BRIP1*	5	0.001688049	23	0.002422309	5	0.000558036	0.696 (0.264–1.834)	0.463	3.032 (0.877–10.487)	0.08
*SLX4*	2	0.000675219	24	0.001277396	8	0.000892857	0.528 (0.125–2.236)	0.386	0.756 (0.160–3.564)	0.724
*ERCC4*	1	0.00033761	13	0.00067353	1	0.000111607	0.496 (0.065–3.792)	0.499	3.026(0.189–48.414)	0.434
Total	38	0.01282917	130	0.012387722	69	0.007700893	–	–		–

*Exploration of breast cancer risk in the East Asian (non-cancer) GnomAD 2.1 population

**Exploration of breast cancer risk in the Westlake Biobank for Chinese (WBBC) population

^a^The number and allele frequency (AF) of Genome Aggregation Database (GnomAD) non-cancer, East Asian subpopulation

## Discussion

Approximately 15–20% of BC cases show familial aggregation or a clear pattern of inheritance (Wendt et al. 2019). In these populations, only a small percentage of patients have detectable pathogenic variants in tumor susceptibility genes (Kurian et al. 2014; LaDuca et al. 2014; Tung et al. 2016). We identified heterozygous mutations in 12 FA genes in 38 of 1481 patients with hereditary high-risk BC in this study. Among them, *FANCA* was the most frequently mutated gene, in agreement with previous findings (Del Valle et al. 2020; Solomon et al. 2015). Pathogenic/likely pathogenic variants in *FANCF*, *FANCR*/*RAD51*, *FANCT*/*UBE2T* and *FANCU*/*XRCC2* were not found in our cohort.

To explore the relationship between FA gene mutations and clinicopathological characteristics, we analyzed pathological findings and clinical data from carriers and non-carriers, which showed significantly more lymph node metastasis in carriers (*p* = 0.041). Larger tumor sizes and lower ER/PR positivity rates were more common among carriers in comparison to non-carriers, although these were not statistically significant. Studies have investigated the association between FA gene expression and BC. Low *FANCD2* expression is related to high histologic grade and pathologic stage in BC (Zhang et al. 2010).Hallajian et al. (2017) found that downregulated expression of *RAD51* was associated with high lymph node involvement in BC. In addition, Wang et al. (2018) reported that high *FANCM* expression was related to low Ki-67 status (*p* = 0.003), and patients with upregulated expression of *FANCM* had better overall survival in luminal B subtype BC. Santarpia et al. (Santarpia et al. 2013) reported that *FANCI* was associated with poor prognosis in ER-positive/HER2-negative BC. After a median follow-up of 38 months, although there was no significant difference in iDFS and DRFS between FA gene mutation carriers and non-carriers in our cohort, the 3.5-year iDFS and DRFS rates tended to be lower in carriers than in non-carriers. These results suggest that loss-of-function variants or downregulated expression of FA genes may be associated with an aggressive phenotype and worse prognosis.

In this study, we evaluated susceptibility to BC for carriers of FA gene mutations and found that *FANCD2*, *FANCM* and *BRIP1* were nearly statistically significant (p = 0.055, 0.08 and 0.08, respectively). *FANCD2* knockout causes animals to develop BC (Houghtaling et al. 2003). Mantere et al. (Mantere et al. 2017) identified that *FANCD2* c.2715 + 1G > A was 2.6-fold more frequent in Finnish BC patients than in controls (*p* = 0.131). In our study, the incidence of *FANCD2* mutation also was trend to more common than that in WBBC controls. The association with BC for *FANCM* mutations has been well investigated, especially for TNBC (Peterlongo et al. 2021). In the Finnish population, *FANCM* c.5101C > T was associated with BC (odds ratio [OR] = 1.86, 95% confidence interval [CI]: 1.26–2.75; *p* = 0.0018), especially with TNBC (OR = 3.56, 95% CI 1.81–6.98, *p* = 0.0002) (Kiiski et al. 2014). However, *FANCM* c.5791C > T was not statistically significantly associated with BC (OR = 1.94, 95% CI 0.87–4.32, *p* = 0.11), but it was associated with increased risk of TNBC (OR = 5.14, 95% CI 1.65–16.0, *p* = 0.005) (Kiiski et al. 2017). Figlioli et al. (2019) reported that *FANCM* c.1972C > T was associated with ER-negative BC and TNBC (OR = 2.44, 95% CI 1.12–5.34, p = 0.034 and OR = 3.79, 95% CI 1.56–9.18, p = 0.009, respectively). In our cohort, *FANCM* showed a trend with increased BC risk (OR = 3.032, 95% CI 0.877–10.487, p = 0.08). These suggest that some FA genes could be candidates for BC susceptibility genes. *BRIP1* was first reported to be associated with BC in 2006 (OR = 2.0, 95% CI 1.2–3.2, *p* = 0.012) (Seal et al. 2006). However, several large-scale studies did not identify *BRIP1* as a BC susceptibility gene (Easton et al. 2016; Hanson et al. 2022; Hu et al. 2021; Weber-Lassalle et al. 2018).

*FANCB* is the only known FA gene on the X chromosome (Kato et al. 2015). Deleterious variants in *FANCB* are rare, and none is registered in the East Asian population of GnomAD and WBBC. Additionally, no pathogenic/likely pathogenic variants in *FANCB* have been reported in BC patients. However, a novel *FANCB* frameshift variant c.1327-3dupT was identified in our cohort. This finding suggests that *FANCB* may be a susceptibility gene for BC.

Additionally, the association between germline mutations in other FA genes (except for *FANCW*/*RFWD3*) and BC risk has been studied. Thompson et al. (Thompson et al. 2012) identified three truncating variants in *FANCC* in 438 familial BC patients that were not found in healthy controls. Palmer et al. (2020) observed moderate risk for African American women carrying *FANCC* mutations with ER-positive BC (OR = 2.42, 95% CI 1.00–5.97, *p* = 0.05). Pan et al. (2019) found that *FANCC* c.339G > A (p.W113X) might contribute to susceptibility in Chinese familial breast and/or ovarian cancer. However, some studies showed different conclusions, that *FANCC* truncation variants (p.R158X and p.R548X) were not associated BC risk (OR = 0.64, 95% CI 0.32–1.29, *p* = 0.215 and OR = 1.03, 95% CI 0.41–2.56, *p* = 0.942, respectively) (Dörk et al. 2019). There was also no significant association between *FANCC* and BC in our cohort. A larger sample may be needed to verify the relationship of mutations in this gene and BC susceptibility.

Several studies have found that polymorphisms in FA genes are relevant to BC risk. *FANCD2* c.4098 T > G (p.Leu1366_Leu1367, rs2272125) was associated with sporadic BC (OR = 1.35, 95% CI 1.09–1.67; *p* = 0.005) (Barroso et al. 2006). In a cohort of Sri Lankan women, *XRCC2* c.*1772G > A (rs3218550) increased the risk of BC (OR = 1.525, 95% CI 1.107–2.101, *p* = 0.0098) (Sirisena et al. 2018). However, *RAD51* c.-1271A > G (rs503078) was found to significantly reduce BC risk (OR = 0.5, 95% CI 0.3–1.0, *p* < 0.05) (Grešner et al. 2020). *ERCC4* c.*971C > G (rs2276466) and other mutations have not been associated with BC risk (Sahaba et al. 2022). In conclusion, germline mutations in FA genes may be related to increased BC risk. Some FA genes may be moderate-penetrance susceptibility genes, and other FA genes have low penetrance. Validation should be performed in studies with larger sample sizes.

PARP inhibitors have been successfully used in patients with breast or ovarian cancer who carry *BRCA1*/*2* germline mutations (Litton et al. 2018; Robson et al. 2017; Weil et al. 2011). TBCRC 048 was an extended study to explore the therapeutic effect of Olaparib monotherapy in metastatic BC with germline or somatic variants in HR-related genes. Mutations in FA genes other than *BRCA1*/*2*, *PALB2* and *RAD51C* were not identified in the study subjects (NCT03344965) (Litton et al. 2018). A clinical study evaluating the efficacy and safety of Olaparib combination immunotherapy in patients with solid tumors carrying HR-related gene mutations is ongoing (NCT04169841) (Fumet et al. 2020). Although conclusive clinical evidence for the utilization of PARP inhibitors in FA gene-mutated cancers is still lacking, a case report provided evidence that one ovarian cancer patient carrying a *FANCA* mutation benefitted from a PARP inhibitor (Qian et al. 2022), which suggests a potential therapeutic option for FA gene-mutated cancers.

There were some limitations in our study. First, this study focused on high-risk hereditary BC cases, which may lead to selection bias in determining the mutation frequencies of FA genes. Second, the median follow-up time was 38 months, and a longer follow-up time is needed to assess prognosis. Third, the number of FA gene mutation carriers was small, so the association between mutations and BC risk could not be confidently evaluated. Fourth, the panel did not cover *FANCV*/*REV7* (Bluteau et al. 2016) and *FANCW*/*RFWD3* (Knies et al. 2017) because this study was designed before they were identified as FA genes.

## Conclusions

To our knowledge, this is the first study to comprehensively investigate FA gene mutations in a relatively large cohort of Chinese BC patients with high genetic risk. This study estimated the prevalence, clinicopathological characteristics, prognosis and risk of BC associated with deleterious variant in FA genes. This exploration enriches our understanding of the role of FA genes in Chinese BC patients. Studies with larger samples are needed to confirm these findings and aid clinical management.

## Data Availability

The data that support the findings of this study are available upon request from the corresponding author.
